# Experimental infection of healthy volunteers with enterotoxigenic *Escherichia coli*wild-type strain TW10598 in a hospital ward

**DOI:** 10.1186/1471-2334-14-482

**Published:** 2014-09-04

**Authors:** Steinar Skrede, Hans Steinsland, Halvor Sommerfelt, Audun Aase, Per Brandtzaeg, Nina Langeland, Rebecca J Cox, Marianne Sævik, Marita Wallevik, Dag Harald Skutlaberg, Marit Gjerde Tellevik, David A Sack, James P Nataro, Anne Berit Guttormsen

**Affiliations:** Division for Infectious Diseases, Department of Medicine, Haukeland University Hospital, N-5021 Bergen, Norway; Department of Clinical Science, University of Bergen, N-5020, Bergen Norway; Department of Biomedicine, University of Bergen, N-5020 Bergen, Norway; Centre for Intervention Science in Maternal and Child Health and Centre for International Health, University of Bergen, PO Box 7804, N-5020 Bergen, Norway; Division of Infectious Disease Control, Norwegian Institute of Public Health, PO Box 4404, Nydalen, N-0403 Oslo, Norway; Laboratory for Immunohistochemistry and Immunopathology (LIIPAT), Centre for Immune Regulation (CIR), University of Oslo, PO Box 4950, Nydalen, N-0424 Oslo, Norway; Department of Pathology, Oslo University Hospital, Rikshospitalet, PO Box 4950, Nydalen, N-0424 Oslo, Norway; Department of Research & Development, Haukeland University Hospital, N-5021 Bergen, Norway; Department of Medical Microbiology, Haukeland University Hospital, N-5021 Bergen, Norway; National Centre for Tropical Infectious Diseases, Department of Medicine, Haukeland University Hospital, N-5021 Bergen, Norway; Department of International Health, Johns Hopkins Bloomberg School of Public Health, Baltimore, 21205 MA USA; Department of Pediatrics, School of Medicine, University of Virginia, P.O. Box 800326, MR4 Room 4012C, 409 Lane Road, Charlottesville, VA 22908 USA; Department of Anesthesia and Intensive Care, Haukeland University Hospital, N-5021 Bergen, Norway; Department of Clinical Medicine, University of Bergen, N-5020 Bergen, Norway

**Keywords:** ETEC, Challenge, Experimental infection, Inpatient

## Abstract

**Background:**

Enterotoxigenic *Escherichia coli* (ETEC) is an important cause of childhood diarrhea in resource-limited regions. It is also an important cause of diarrhea in travellers to these areas.

To evaluate the protective efficacy of new ETEC vaccines that are under development, there is a need to increase the capacity to undertake Phase IIB (human challenge) clinical trials and to develop suitable challenge models.

**Methods:**

An in-hospital study was performed where fasting adult volunteers were experimentally infected with 1 × 10^6^ to 1 × 10^9^ colony forming units (CFUs) of the wild-type ETEC strain TW10598, which had been isolated from a child with diarrhea in West Africa in 1997. We recorded symptoms and physical signs and measured serum immune response to the TW10598 bacterium.

**Results:**

We included 30 volunteers with mean age 22.8 (range 19.8, 27.4) years. The most common symptoms were diarrhea (77%), abdominal pain (67%), nausea (63%), and abdominal cramping (53%). Seven subjects (23%) experienced fever, none were hypotensive. Most of the volunteers responded with a substantial rise in the level of serum IgA antibodies against the challenge strain.

**Conclusions:**

We established the capacity and methods for safely undertaking challenge studies to measure the efficacy of ETEC vaccine candidates in a hospital ward. Strain TW10598 elicited both clinical symptoms and an immune response across the doses given.

**Electronic supplementary material:**

The online version of this article (doi:10.1186/1471-2334-14-482) contains supplementary material, which is available to authorized users.

## Background

A recent case–control study on the burden and etiology of moderate to severe diarrhea in children in low- and middle-income countries (LMICs) demonstrated that ETEC remains among the most important bacterial pathogens and that ST-producing ETEC infection was associated with increased case fatality [1]. There is a need for a broadly protective ETEC vaccine [2], but a number of significant barriers must be overcome before an effective vaccine will be available [2, 3], and despite four decades’ of focused efforts, no effective ETEC vaccine has been developed. However, there is hope that effective vaccines may be developed, because natural ETEC infections appear to protect against new infections [4, 5].

Part of the challenge of developing effective ETEC vaccines is that, without considerable manipulation, human ETEC do not effectively colonize and induce diarrhea in animals. Human studies are needed to evaluate the immunogenicity and protective efficacy of new vaccines. Human experimental infection and challenge studies are typically performed in dedicated facilities that can house 10–20 volunteers under “enteric precaution”, in Norway termed “in isolation” during the phase when the volunteers are still infectious. To date the total number of study subjects included in experimental ETEC infection studies is only about 400 [6]. Undertaking such studies is resource-demanding and few, often highly specialized, facilities can perform them. This complicates the evaluation of new ETEC vaccine candidates [7].

In human vaccine challenge studies (Phase IIB trials), volunteers are usually allocated to receive a vaccine or placebo, followed by experimental infection with a live wild-type ETEC strain after 2–3 months. The ETEC strains most commonly used in such studies include H10407, B7A, and E24377A [6]. These strains may not, however, be good representatives for the ETEC types most commonly causing diarrhea in LMIC children. Strain H10407 has the rare ability of expressing all three ETEC toxins (human heat-stable toxin (STh), porcine heat-stable toxin (STp), and LT. Strain B7A has a rare toxin-colonization factor combination (STh LT-Coli Surface antigen 6 [CS6]), while E24377A appears to have an ancestral origin that differs from other SThLT-CS1 CS3 strains [8].

To establish a human challenge model with a new ETEC strain for use in clinical trials, there is first a need to do an experimental infection study to ensure the safety of ingesting the strain, and to determine the dose that would induce a reliably high attack risk while minimizing the likelihood of overwhelming an otherwise protective immune response. To increase the capacity to perform Phase IIB trials with human ETEC vaccines, and to introduce a new challenge model for such studies, we undertook an experimental infection study in a hospital ward at Haukeland University Hospital (HUH), Bergen, Norway, with an epidemiologically relevant wild-type ETEC strain previously not used to experimentally infect volunteers.

## Methods

### Study subjects

Healthy 18–40 year-old subjects who had not traveled to LMICs for the past 9 months were recruited by oral and written information presented in a step-wise manner. Two investigators informed potential volunteers in plenary sessions. Those interested were given further individualized in-depth oral and written information. Following a final interview, subjects were required to complete a written questionnaire to ensure that they had understood the study rationale, potential benefits and risks, and what procedures that were to be conducted. We then obtained written informed consent. Primary targets for this information were students at the University of Bergen.

### Challenge strain

Following a comprehensive phylogenetic analysis of human ETEC isolates [8, 9], we selected strain TW10598 because it is a good representative for one of the major ETEC ancestral lineages that are often associated with childhood diarrhea [8].

The O6:K15:H16 strain was isolated in 1997 from a 14 month old girl in Guinea-Bissau suffering from diarrhea [10], and it is capable of producing STh and LT, as well as the ETEC colonization factors CS2, CS3, and CS21. It is sensitive to ciprofloxacin and trimethoprim-sulfamethoxazole but resistant to ampicillin. A single-colony pick of TW10598 had undergone genome sequencing (GenBank BioProject ID: PRJNA59743) [9], and a master cell bank and subsequent working cell bank (WCB) were produced from this culture by the Inoculum Preparation Laboratory of the Center for Vaccine Development at the University of Maryland School of Medicine. Frozen WCB vials containing high-density bacterial growth were shipped on dry-ice to HUH and checked for the presence of ST and LT genes by PCR [11] before being stored at -80°C in a temperature-monitored freezer.

### Setting

The study was conducted between 29^th^ of March 2011 and 31^st^ of May 2013 in the Division for Infectious Diseases (ID) at Department of Medicine at HUH, Bergen. HUH is a 1,100-bed teaching hospital serving a population of approximately 280,000 inhabitants. It is also a referral hospital for almost 1 million people in Western Norway. The ID division runs two patient wards with altogether 31 beds, 12 of which are in 10 cohort isolation rooms. The volunteers were admitted primarily at times of low prevalence of communicable diseases in the local population.

### Ethics approval

The study was approved by the Regional Committee for Medical and Health Research Ethics, Health Region West (REC-West, case number 2010/728-14).

### Volunteer enrolment

We screened all eligible volunteers in the outpatient clinic a median 7 (Range: 4, 46; Interquartile range: 6, 11) days prior to infection, recorded their personal data and medical history, and undertook a physical examination. Blood specimens were collected five times from all subjects: At the screening visit, within 24 hours of challenge (day 0), and at days 7, 10, and 28. The specimens were used for total blood cell counts and measurement of serum concentrations of electrolytes, creatinine, alanine aminotransferase (ALT), and glucose. Serum antibodies to hepatitis B and hepatitis C virus were measured, and a combined antibody/antigen assay for HIV infection was undertaken at the screening visit. Urine tests, including a pregnancy test in women, were performed, and an electrocardiogram was taken. Stool specimens were collected for examination for occult blood in feces (Hemo-Fec^®^, Diag Nor AS, Asker, Norway). All subjects were screened for an array of enteropathogens in feces. The inclusion criteria were: a signed informed consent, age between 18 and 40, completed health questionnaire, stool culture without enteropathogens, normal base-line blood tests, being able to be in isolation for up to 10 days, and effective contraception in the women. The exclusion criteria were: fever (≥38°C) during the last 48 hours before study initiation, participation in another clinical trial during the last 3 months, use of an immunosuppressive agent, pregnancy, breastfeeding, and positive fecal occult blood test. In addition, subjects were excluded from the study if they had a history of any chronic gastrointestinal conditions. From the day of experimental infection, the volunteers were hospitalized. The volunteers were admitted to the Infectious Diseases ward one or two at a time, and stayed in a cohort isolation room suited for the study. The volunteers and the study personnel adhered strictly to the hospital infection control procedures.

### Preparation of inocula

The inocula and the necessary media and buffers were prepared by following a study-specific procedure. All steps of the operator’s preparations were followed by at least one observer to minimize the risk of errors. Bacterial cultures were based on reagents free of animal products. Material from WCB vials was streaked onto Luria-Bertani (LB) agar, and six colonies apparent after over-night incubation at 37°C in a temperature-monitored regular incubator were mixed together in phosphate-buffered saline (PBS) and pipetted onto LB agar dishes to make a lawn. After over-night incubation at 37°C, bacterial cells were scraped from the agar surface and resuspended in PBS. The bacteria were then pelleted by centrifugation at 2,000 × g for 5 min and resuspended in PBS. This procedure was repeated three times. Cell density of the resulting stock solution was determined by measuring the absorbance at 600 nm with a spectrophotometer and comparing the absorption with a pre-determined reference curve. Based on these results, aliquots were subsequently diluted in PBS to provide the correct bacterial cell concentration, and thereby volume for the required dose. Before and after administering the dose, an aliquot of the inoculum was serially diluted in PBS and plated in triplicate onto LB agar, and the actual dose given was back-calculated from the colony counts following over-night incubation at 37°C.

### Experimental infection

The volunteers fasted overnight, only being allowed to drink water after midnight the day before challenge. One minute before receiving the challenge strain, the subjects drank 120 ml 1.33% bicarbonate buffer to neutralize gastric acid. The dose of strain TW10598 was suspended in 2 ml PBS, to which 30 ml bicarbonate buffer was added just before drinking. The volunteers were allowed to eat and drink normally 1 hour after ingesting the dose. The first volunteers were given a low (1 × 10^6^ CFU) dose, and at least three volunteers received a given dose before we decided whether to increase the dose 10-fold for the next group of volunteers. The decision to increase the dose was based on estimates on diarrheal attack risk defined as the percentage of volunteers who got diarrhea, as well as an assessment of their symptoms.

To clear the infection, the volunteers were given orally 2 × 500 mg ciprofloxacin daily for three days. This treatment is generally used in ETEC challenge studies [12], and is recommended by the Infectious Diseases Society of America (IDSA) [13]. Indications for early initiation of treatment were mild or moderate diarrhea accompanied by clinical signs of dehydration or malaise, or moderate or severe diarrhea according to definitions presented below. Also, a volunteer would receive early treatment if the study clinician decided antibiotic treatment was indicated. If none of these criteria were fulfilled, the volunteer would receive ciprofloxacin from day 5.

Microbiological analyses of stool cultures were performed daily. The challenge strain was detected from stool specimens using PCR for the ST and LT genes. Stool specimens were plated on Lactose agar for the identification of *E. coli*. After aerobic incubation overnight at 35°C, a representative selection of the bacteria were collected by swabbing the confluent part of the agar plate with a 1 μl inoculating loop followed by suspension in 0.5 ml distilled water. The sample was boiled for 10 minutes followed by centrifugation at 16,000 × g for 5 minutes. Five μl of the supernatant was used as PCR template and amplified in a final volume of 20 μl reaction mixture containing LightCycler^®^ FastStart DNA Master^PLUS^ SybrGreen I (Roche Diagnostics GmbH, Germany) and 0,5 μM of each primer. The primers used in this study were ST-gene_forward_ (JW7): 5′-CAC-CCG-GTA-CAR-GCA-GGA-TT-3′; ST-gene_reverse_ (JW14): 5′- ATT-TTT-MTT-TCT-GTA-TTR-TCT-T-3′; LT-A-gene_forward_ (TW20): 5′- GGC-GAC-AGA-TTA-TAC-CGT-GC-3′; and LT-A-gene_reverse_ (JW11): 5′-CGG-TCT-CTA-TAT-TCC-CTG-TT-3′ [11]. PCRs were performed using a LightCycler^®^ 2.0 (Roche Diagnostics) instrument. An initial pre-incubation step at 95°C for 10 minutes was followed by 35 cycles of denaturation at 95°C for 10 seconds, annealing at 55°C for 10 seconds and extension at 72°C for 10 seconds. After amplification, melting curve analysis at 65°C to 95°C was performed with a temperature transition rate of 0.1°C/second to determine the melting temperature (T_m_) values for amplified PCR products. A Tm of 79.0 ± 0.5°C and 82.0 ± 0.5°C was considered to indicate the presence of the STh and LT-A gene, respectively. A negative control (distilled water) and a positive control (extracted DNA from the challenge strain were included in every PCR run. The efficiency of the ST-gene amplification was sometimes low. For this reason, we considered a stool specimen positive for strain TW10598 if the ST and/or the LT-A gene was detected.

The isolation of a volunteer was suspended when 3 consecutive stool specimens were negative for ETEC. A follow-up examination in the out-patient clinic was performed at day 28, and a phone call at day 84 terminated the follow-up period.

### Clinical evaluation

During the period of hospitalization, volunteers were interviewed and examined by a study physician once daily. Study nurses and other clinical staff at the ward assessed the volunteers a minimum of three times a day. Observations were documented by use of individual Case Report Forms. Blood pressure, heart rate, and oxygen saturation was assessed by Criticare eQuality Vital Signs Monitor (Criticare Systems Inc., Waukesha, WI, USA), and body temperature was measured with the Light Touch LTX infrared thermometer (Exergen Corp., Watertown, MA, USA).

Nausea, vomiting, abdominal pain or cramping, urgency, bloating, flatulence, decreased appetite, constipation, fever, chills, malaise, myalgias, headache, lightheadedness and any organ system-related symptom were recorded and graded daily as described elsewhere [12]. Symptoms were considered elicited by the ETEC infection if they occurred after challenge and before the end of the day the volunteer received the first dose of ciprofloxacin.

Evaluation of stools and diarrhea was performed essentially as described elsewhere [12].

The volunteers were considered to have diarrhea when they passed 1 loose stool (≥grade-3) of ≥300 g or ≥2 loose stools (≥grade-3) totaling ≥200 g during any 48-hour period. In cases where grade-1 or grade-2 stools were observed within a diarrheal episode, we did not consider these to represent diarrheal stools. If a volunteer experienced two separate diarrheal episodes, this was considered to represent a single, continuous episode. For any diarrhea episode defined by a single diarrheal stool, the diarrhea duration was set to one hour. Diarrhea was deemed to be mild in cases where, during any 24 hour period of the episode, the subject experienced 1 to 3 diarrheal stools with a total weight of ≤400 grams; moderate for 4 or 5 diarrheal stools, or a total weight of 401–799 grams; and severe for ≥6 diarrheal stools, or a total weight of ≥800 grams. Diarrhea would also be considered severe in cases in need of intravenous fluid treatment. If severity changed during an episode, we reported the most severe grade.

### Serum IgA antibodies against ETEC

Serum specimens were collected on days 0, 7, 10, and 28, and frozen at -80°C until it was used to measure IgA directed against the challenge strain. Serum antibodies against strain TW10598 were measured by flow cytometry with live bacteria [14], which were grown overnight, harvested, washed in Hank’s balanced salt solution with bovine serum albumin (HBSS/BSA), and adjusted to an OD_650 nm_ of 0.65 in HBSS/BSA. A serum specimen from one of the participants with a high serum IgA antibody level against strain TW10598 was included in each plate to generate a standard curve. Fifty microliters of serum at various dilutions in HBSS/BSA and 5 μl of bacterial suspension were mixed by pipetting and incubated for 45 minutes at 37°C. They were then washed twice with HBSS/BSA by centrifugation (850 × g for 3 min) and incubated with anti-IgA R-phycoerythrin-(PE)-conjugate goat anti-human IgA (2050–09, Southern Biotech, Birmingham, AL, USA) at 1/100 in HBSS/BSA for 45 minutes. Finally, the bacteria were washed and suspended in 100 μl HBSS/BSA supplemented with 10 μg/ml of the DNA-binding fluorescent dye Hoechst 34580. The fluorescence intensity reflecting bound antibodies was read by an Attune flow cytometer (Life Technologies, Carlsbad, CA, USA), gating on the bacteria with combined side scatter/Hoechst 34580 fluorescence. The standard curve was drawn from the geometric mean PE fluorescence intensity (GMFI) of the various dilutions of the standard serum which was assigned a value of 1,000 arbitrary units (AU)/ml. The GMFI from the specimens were interpolated on the standard curve with GraphPad Prism software version 5 (GraphPad Software, Inc., San Diego, CA). We calculated geometric mean serum level of IgA directed against the challenge strain and the fold-increase in geometric mean titers from day 0 to day 7, 10 and to 28.

## Results

### Volunteer characteristics

Thirty-two volunteers agreed to participate in the study. Two subjects were excluded: one had an upper respiratory tract infection with fever the day before the intended ETEC challenge, whereas asymptomatic infection with *Campylobacter jejuni* was detected in the other volunteer. The volunteers entered the study in 21 separate groups. Of the 21 female and 9 male volunteers, 28 were medical students. Their mean age was 22.8 years old (range: 19.8, 27.4; standard deviation: 1.95), and their body mass indices ranged from 18.3 to 42.5 with a median 22.1 (interquartile range 20.7, 25.3) kg/m^2^.

### Clinical response

The target doses for this study were 1 × 10^6^, 1 × 10^7^, 1 × 10^8^, and 1 × 10^9^ CFU, while the actual doses given had ranges 0.9–1.0 × 10^6^, 0.7–1.4 × 10^7^, 0.62–1.4 × 10^8^, and 0.82–1.5 × 10^9^ CFU, respectively. Twenty-three volunteers (77%) developed diarrhea, of which five had mild, nine moderate, and nine severe episodes (Table 1). The median severity was mild to moderate, moderate, moderate to severe, and moderate for those who received 1 × 10^6^, 1 × 10^7^, 1 × 10^8^, and 1 × 10^9^ CFU of strain TW10598, respectively.

**Table 1:** **Proportion of subjects with diarrhea, incubation period, stool output and episode duration among 30 volunteers experimentally infected with ETEC strain TW10598 (STh LT-CS2 CS3 CS21; O6:K15:H16)**

Target dose (CFU)	No. of volunteers	No. with diarrhea	Attack risk	Median severity	Mean incubation period, hours (range)	Mean 24 hrs maximum stool output, grams (range)	Mean whole episode stool output, grams (range)	Mean episode duration, hrs (range)	Mean 24 hrs maximum stool output, count (range)
1 × 10^6^	3	2	67%	Mild-Moderate	60 (48–72)	416 (330–502)	613 (510–717)	37 (25–48)	2 (2–2)
1 × 10^7^	8	5	63%	Moderate	37 (9–70)	676 (407–1352)	877 (407–1842)	38 (6–72)	5 (3–9)
1 × 10^8^	7	6	86%	Moderate-Severe	38 (13–78)	965 (318–1790)	1466 (450–3608)	46 (18–78)	6.5 (3–13)
1 × 10^9^	12	10	83%	Moderate	30 (10–57)	538 (295–918)	687 (358–1315)	33 (1–106)	4.2 (1–9)

The mean incubation period, i.e. the period from challenge to the debut of symptoms, was 60 hours among those receiving 1 × 10^6^ CFU, and between 30 and 38 hours for those receiving higher doses. Diarrhea duration ranged from 1 to 106 hours in the volunteers. The number of bowel movements during diarrheal episodes ranged from 1 to 13, and stool weight ranged from 295 to 1,790 grams per 24 hours. While nurses provided necessary fluids to prevent dehydration, the study physicians decided that none of the volunteers required rehydration with oral rehydration salts solution or with intravenous fluids. ETEC was detected in stool specimens of all volunteers prior to ciprofloxacin treatment.

The most frequent symptoms other than diarrhea were nausea, abdominal pain, abdominal cramping, headache, malaise, and decreased appetite (Table 2). Most symptoms were grade-1 and 2, none of the volunteers had grade-4 or -5 symptoms, but two volunteers had grade-3 abdominal pain, 1 had grade-3 abdominal cramps, and one had grade-3 bloating. There were seven cases with fever (all grade-1), and six with chills (5 grade-1 and 1 grade-2), but no case with signs of hypovolemia. No severe adverse events were observed.

**Table 2:** **Symptoms and signs other than diarrhea in 30 volunteers experimentally infected with ETEC strain TW10598**

Target dose (CFU)	1 × 10^6^	1 × 10^7^	1 × 10^8^	1 × 10^9^
No. of volunteers	3	8	7	12
Nausea	1 (33%)	5 (63%)	5 (71%)	7 (58%)
Abdominal pain	0	4 (50%)	7 (100%)	7 (58%)
Abdominal cramping	0	1 (13%)	7 (100%)	6 (50%)
Excessive flatus	1 (33%)	1 (13%)	5 (71%)	5 (42%)
Decreased appetite	0	2 (25%)	5 (71%)	5 (42%)
Bloating	1 (33%)	0	0	6 (50%)
Vomiting	0	1 (13%)	0	2 (17%)
Constipation	0	0	0	1 (8%)
Headache	2 (67%)	2 (25%)	4 (57%)	5 (42%)
Malaise	0	2 (25%)	4 (57%)	5 (42%)
Fever	0	4 (50%)	1 (14%)	2 (17%)
Chills	0	1 (13%)	1 (14%)	4 (33%)
Myalgias	0	1 (13%)	0	4 (33%)
Lightheadedness	0	2 (25%)	0	1 (8%)
Hypovolemia	0	0	0	0

### Serum anti-TW105098 IgA response

**Figure 1:**
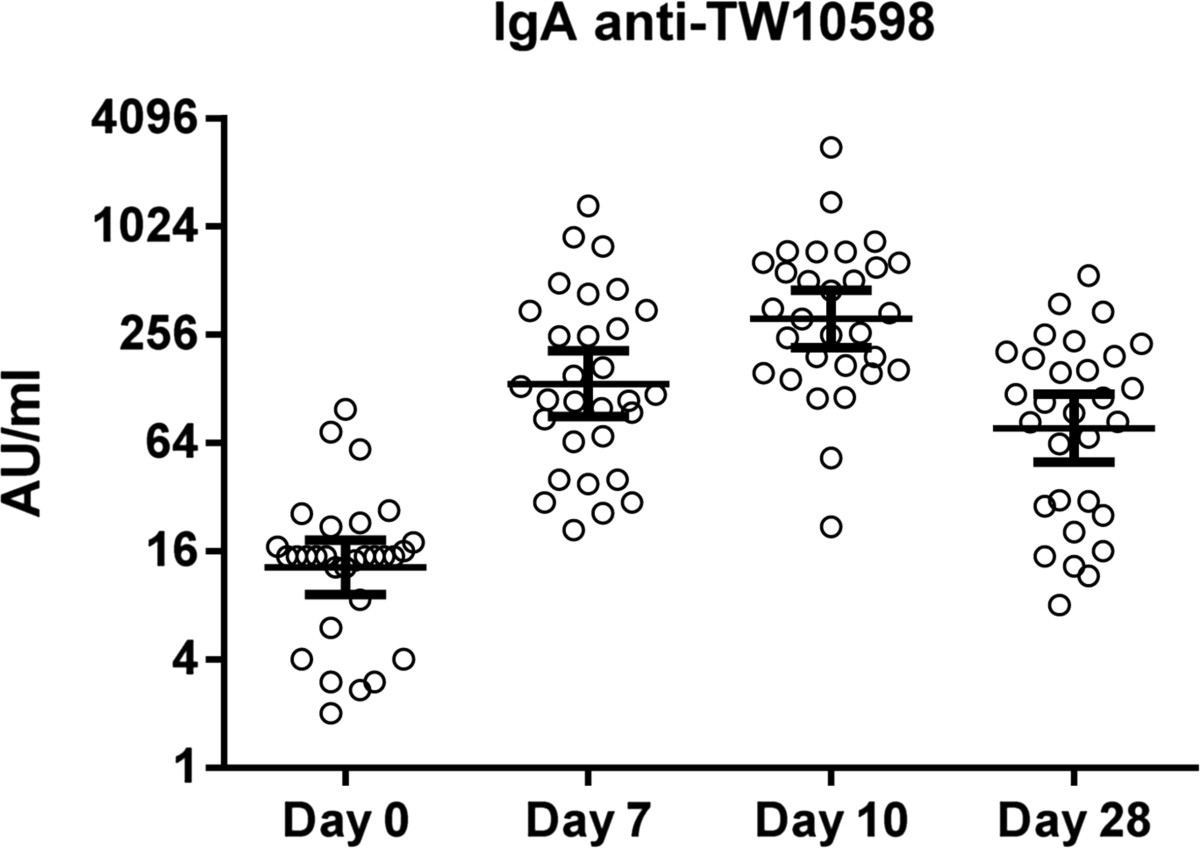
**Serum IgA antibody levels against the TW10598 ETEC challenge strain.** Serum IgA antibody levels against live TW10598 bacteria measured as arbitrary units (AU) by flow cytometry in 30 adult volunteers at different time points, pre (day 0) and post experimental infection. Circles represent the IgA antibody level of each volunteer; horizontal lines indicate geometric means and 95% confidence intervals.

In the pre-challenge sera (day 0), the geometric mean level (GML) of IgA antibodies against TW10598 was 13.0 AU/ml (CI: 9.2, 18.5) (Figure 1). On day 7, 10 and 28 after challenge, the corresponding IgA antibody levels were 136.8 AU/ml (CI: 90.0, 208.0), 314.4 AU/ml (CI: 217.9, 453.7) and 77.3 AU/ml (CI: 49.9, 119.7), respectively. All but one participant exhibited at least a two-fold response to the challenge strain. The low-responding volunteer received a dose of 1 × 10^7^ CFUs, did not develop diarrhea, and was the only volunteer who did not experience any other symptoms from the infection. The geometric mean fold increase in the IgA level from day 0 to day 7, 10, and 28 was 10.5 (CI: 6.5, 16.9), 24.1 (CI: 15.2, 38.1), and 5.9 (CI: 3.7, 9.5), respectively.

## Discussion

In the present study, there were two specific aims that may contribute to the development of ETEC vaccines. First, as there is a lack of sites that are capable of performing large experimental infection studies, including Phase IIB trials, and, to the best of our knowledge, no facility for such studies in Scandinavia, we aimed at establishing such capacity at HUH. We undertook the study in the hospital in a way that was compatible with everyday work in the ID ward, and the method should be subsequently applicable in similar institutions elsewhere. The second aim was to develop a new challenge model for ETEC, involving an epidemiologically relevant strain.

The present work describes the methods used to establish competence and capacity to perform experimental infection with ETEC within our hospital. HUH is co-located with the University of Bergen, and, in our setting, we chose to approach students, many of them studying to become physicians. We believe these students were likely to adequately comprehend all information required to refuse or provide a genuinely informed consent, as well as follow the study procedures. As HUH has a restricted number of beds under isolation, we had to rely on inclusion of a few individuals at a time. This was possible in our student population, as we could adjust their admission according to the availability of cohort isolation rooms.

It may be challenging to free cohort isolation rooms in a hospital such as ours. This problem was overcome by meticulous planning and administration of their use. Thus, volunteers were admitted primarily at times of low prevalence of communicable diseases, such as influenza, in our community.

The study team ensured volunteer safety by careful clinical monitoring similar to that presented in studies taking place in dedicated units and prompt treatment using procedures already established for care of patients with severe invasive and non-invasive infections. The study principal investigator (ABG) is a senior intensive care specialist and responsible for the evaluation of the volunteers on a daily basis. Although in our study there was no need for any advanced treatment modalities, the safety level was probably equal to or higher than that offered in most dedicated research units.

We found that our approach of undertaking the study in a hospital ID ward was safe for the volunteers, and should be applicable to other hospitals that lack research facilities dedicated to challenge studies. It should be noted, however, that the approach probably is less efficient and more costly and time-consuming than performing similar studies in such facilities. Further, while our approach is suitable for establishing the safety and dose of experimental infection with a given ETEC strain, a dedicated unit would be required to undertake a phase IIB vaccine trial.

The second goal of our study was to develop a new challenge model that could be used in future Phase IIB trials for testing ETEC vaccine prototypes. For our model, we elected a strain that has phenotypic traits and an ancestral origin common to ETEC strains often found among LMIC children with diarrhea [8]. In the present study, we found that experimental infection with TW10598 was safe and gave mild systemic symptoms and signs. We also found that the strain was capable of eliciting a strong immune response, in that most volunteers responded with a >20-fold increase in serum IgA anti-TW105098 level from day 0 to day 10. The response appeared to be strain specific because the volunteers did not respond to ETEC strain H10407, which is ancestrally closely related to TW10598 [8] but expresses a different serotype and ETEC colonization factor (data not shown).

In our study we administered doses used in other ETEC challenge studies, i.e. 1 × 10^6^ to 1 × 10^9^ CFUs. In their review of experimental infection, Porter *et al.*[6] found that when limiting their analyses to the three most used ETEC strains (H10407, B7A, and E24377A) the diarrhea attack risk was dose-dependent. For these strains they found no difference in diarrhea attack risks across any of the strains at doses of 5 × 10^8^ CFUs up to 1 × 10^10^, with an overall attack risk of 87%. For a Phase IIB trial, we recommend using a dose of 1 × 10^8^ CFU, which gave an 86% attack risk in our study. This dose is similar to what is normally used in ETEC challenge studies [6]. From Table 1, it may seem like volunteers who were given 10^8^ CFU had more severe diarrhea (frequency and volume) than those who were given 10^9^. However, the observations are based on data from only 16 volunteers, and the differences are not statistically significant (data not shown).

We found that most of the volunteers responded to the infection with a substantial rise in the level of serum IgA antibodies against the challenge strain. This is comparable to results obtained with LPS-based ELISA after challenge with H10407 [12]. We used a method based on flow-cytometry because it is faster than ELISA, and it produces a better signal–to-background ratio with fewer spurious readings.

Compared to other studies aimed at developing a new ETEC challenge model, the number of included volunteers is high. The reason for this is that we wanted to estimate the attack risk and confirm the safety of the experimental infection and use the data and specimens collected during the study for further studies of immune responses to ETEC infections.

## Conclusions

We describe a novel in-hospital experimental ETEC infection study in healthy volunteers, in which ETEC strain TW10598 elicited diarrhea, abdominal and constitutional symptoms, and induced strong strain-specific immune responses. The experimental infection was safe, elicited moderate symptoms, and methods and capacity needed to undertake Phase IIB trials were established.

## Authors’ original submitted files for images

Below are the links to the authors’ original submitted files for images. Authors’ original file for figure 1

## Abbreviations

ALT: Alanine aminotransferase
CFU: Colony forming unit
CS: Coli surface antigen
ETEC: Enterotoxigenic *Escherichia coli*
HUH: Haukeland University Hospital
ID: Infectious diseases
IgA: Immunoglobulin A
LB-agar: Luria-Bertani agar
LMIC: Low- and middle-income countries
LT: Heat-labile toxin
PBS: Phosphate-buffered saline
PCR: Polymerase chain reaction
STh: Human heat-stable toxin
STp: Porcine heat-stable toxin
WCB: Working cell bank.
