# Supplementary figures and images for: Experimental infection of healthy volunteers with enterotoxigenic Escherichia coliwild-type strain TW10598 in a hospital ward
Source: BMC Infect Dis. 2014 Sep 4;14:482. doi: 10.1186/1471-2334-14-482 (PMC4165915; doi:10.1186/1471-2334-14-482)

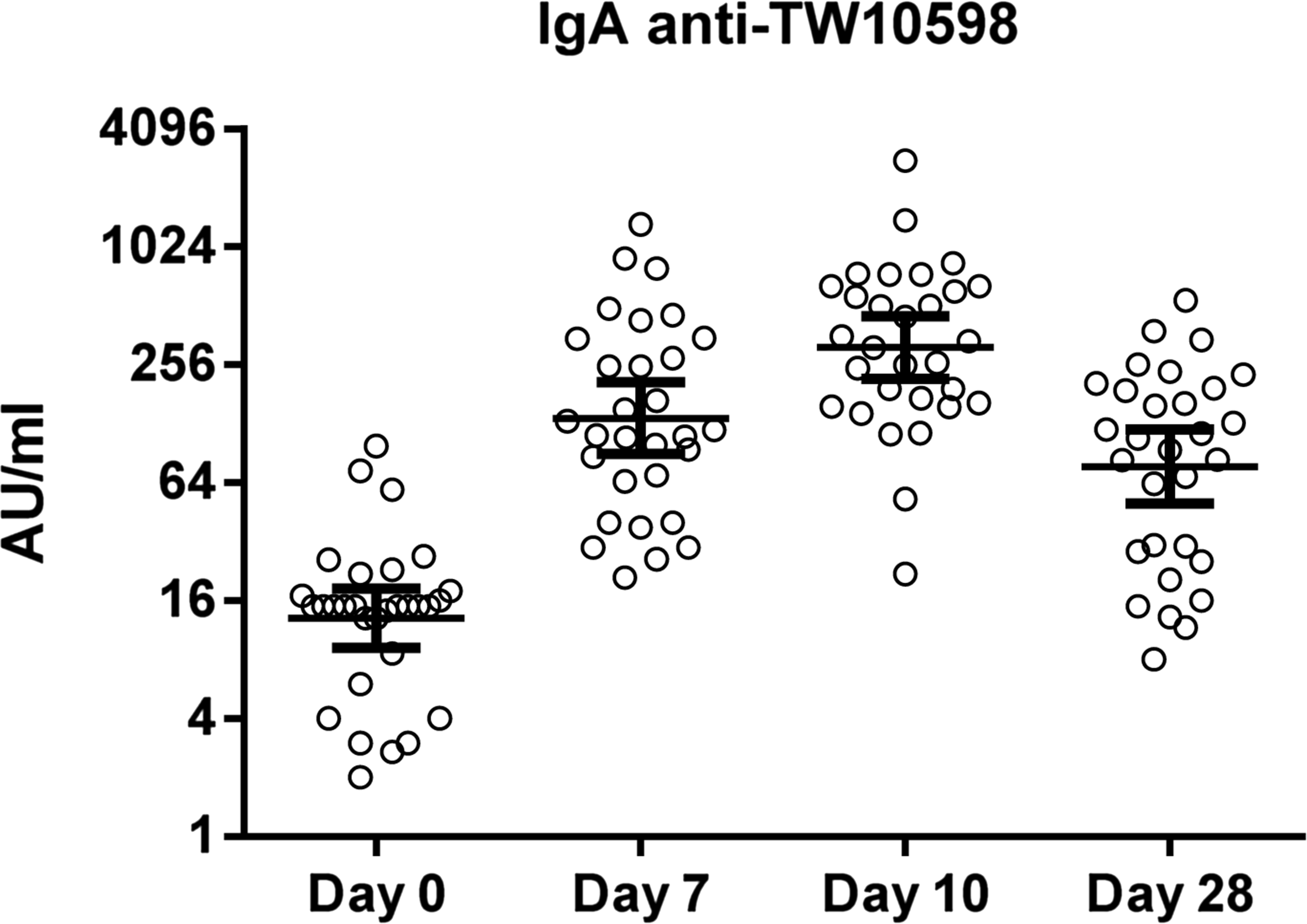

Supplement: Supplementary file 1 — Authors’ original file for figure 1 [file 12879_2014_3794_MOESM1_ESM.tif]
